# Predictive Simulation Generates Human Adaptations during Loaded and Inclined Walking

**DOI:** 10.1371/journal.pone.0121407

**Published:** 2015-04-01

**Authors:** Tim W. Dorn, Jack M. Wang, Jennifer L. Hicks, Scott L. Delp

**Affiliations:** 1 Department of Bioengineering, Stanford University, Stanford, California, United States of America; 2 Department of Computer Science, University of Hong Kong, Hong Kong, China; 3 Department of Mechanical Engineering, Stanford University, Stanford, California, United States of America; Delft University of Technology (TUDelft), NETHERLANDS

## Abstract

Predictive simulation is a powerful approach for analyzing human locomotion. Unlike techniques that track experimental data, predictive simulations synthesize gaits by minimizing a high-level objective such as metabolic energy expenditure while satisfying task requirements like achieving a target velocity. The fidelity of predictive gait simulations has only been systematically evaluated for locomotion data on flat ground. In this study, we construct a predictive simulation framework based on energy minimization and use it to generate normal walking, along with walking with a range of carried loads and up a range of inclines. The simulation is muscle-driven and includes controllers based on muscle force and stretch reflexes and contact state of the legs. We demonstrate how human-like locomotor strategies emerge from adapting the model to a range of environmental changes. Our simulation dynamics not only show good agreement with experimental data for normal walking on flat ground (92% of joint angle trajectories and 78% of joint torque trajectories lie within 1 standard deviation of experimental data), but also reproduce many of the salient changes in joint angles, joint moments, muscle coordination, and metabolic energy expenditure observed in experimental studies of loaded and inclined walking.

## Introduction

Creating models of human locomotion that synthesize gait patterns in the absence of experimental data—often called predictive simulation—is a fundamental problem in biomechanics. Predictive simulation uses high-level specifications, such as achieving a target velocity and minimizing metabolic cost, to synthesize full-body dynamics, typically by applying optimization under physical and physiological constraints [1–3]. The ability to synthesize human-like motion from minimal experimental inputs has a wide range of potential uses, including analyzing gait pathologies, designing assistive devices, and planning musculoskeletal surgeries. To employ predictive gait simulations in these applications, methods must accurately predict changes in parameters of interest, such as joint angles and torques, muscle activations, and metabolic energy expenditures for a range of locomotor activities.

Musculoskeletal simulations are usually developed to track experimental measurements and then used to estimate quantities that are difficult or impossible to measure experimentally [4–8]. For example, simulation-based estimates of muscle forces have revealed which muscles are responsible for body weight support and forward progression during level walking and running [9–11]. Tracking-based simulation approaches have also revealed how muscles forces affect internal joint loads [12]. Although simulation methods that rely on tracking a priori experimental data have offered valuable insights into human movement, these methods cannot be used to predict new motions for novel tasks or environmental conditions where existing data is not available. Predictive simulations are capable of predicting new movements, as they do not explicitly track motion data, and can also reveal muscle forces and other hard-to-measure quantities for these novel motions.

The most widely-employed framework for predictive simulation synthesizes motion by optimizing kinematic, joint torque, or muscle excitation trajectories that minimize some measure of effort while satisfying high-level task requirements [13–21]. This paradigm, also called optimal control, is valuable in the study of human locomotion because new motions can be synthesized as the surrounding environment is changed. Anderson and Pandy [17] synthesized a three-dimensional muscle-driven cycle of gait that reproduced the features of human walking by computing the pattern of activation for 54 muscles that minimized metabolic energy. However, the optimization was prohibitively expensive due to the high dimensionality of the muscle excitation trajectories and the necessity to accurately integrate the equations of motion. Ackermann and van den Bogert showed that direct collocation methods [22], which avoid integration altogether, can be used to study human walking [19] and low-gravity skipping [23] and are much more computationally efficient. Miller et al. employed predictive optimization to investigate limits of sprinting speed [20], as well as gain insights into the chosen objective terms [24] and muscle energy model [25]. Alternatively, Neptune et al. [7] synthesized optimal walking in a lower dimensional space constructed from muscle synergies, which were computed from experimental normal walking data. Each of the above studies synthesized motion by discretizing each muscle excitation trajectory into individual nodes over time and optimizing for the value of these nodes. The resulting trajectories do not change based on feedback from the musculoskeletal model or the environment, and would fail to take a new step even under minor differences in external forces. Through a combination of CPG (central pattern generator) inspired control architecture and environmental feedbacks, Taga et al. [15, 16] and Ogihara and Yamazaki [18] demonstrated that more robust controllers can be constructed. More recently, Geyer and Herr [26] proposed a set of simple muscle control laws based on specific force and length reflexes that were able to generate the complex neural excitation signals for human locomotion. The control parameters constitute a low-dimensional representation of muscle excitations and can be optimized to generate a variety of gaits under different environments, as demonstrated by Wang et al. [27].

There is a long history of work on predicting biological motion using optimization [1, 28, 29] including gait [13, 30]. While significant progress has been made over the years—including simulating gaits in perturbed environments [16, 23, 25, 26, 31]—systematic evaluations against experimental data have been largely limited to normal, level walking. Comparisons to independent ground truth data beyond a single nominal motion are crucial for properly evaluating the reliability and flexibility of predictive simulation methods.

The goal of our study was to demonstrate that a single predictive simulation model can be used to simulate human walking movements that are comparable to ground truth data in a variety of environments—specifically, while carrying different loads on the back and over different inclines. Our first aim was to synthesize a dynamic simulation of normal human walking that reproduced the kinematics, kinetics, and muscle coordination observed in gait experiments, building on the approach of Wang et al. [27] and Geyer and Herr [26]. Our second aim was to introduce a range of backpack loads, ground inclines, and generate simulations that predict the salient gait adaptations reported in experimental studies [32–35].

## Methods

**Fig 1:**
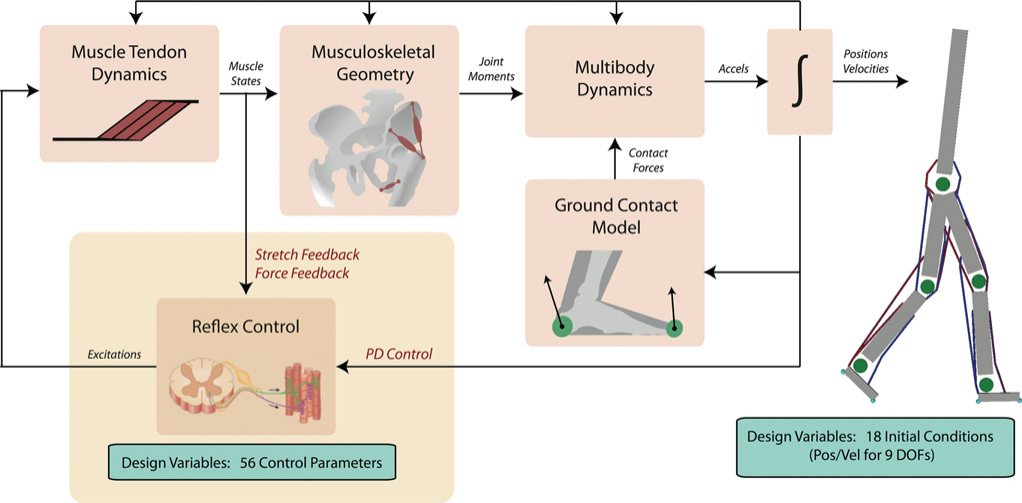
The model and optimization framework for simulating locomotion. The OpenSim musculoskeletal model has 9 degrees of freedom and 8 musculotendon actuators per leg. The dynamics of each musculotendon actuator are governed by force-length-velocity properties and first order excitation-activation dynamics. Ground contact is represented by two compliant spheres on each foot with Coulomb friction. The model’s controllers include stretch and force feedback, as well as proportional-derivative control. The parameters of these controllers and 18 initial position and velocities of the model constitute design variables that we optimized to generate simulations of locomotion.

We developed a planar, muscle-driven dynamics model to simulate gait (Fig. 1). The model was controlled by a set of reflex-based control laws that generate muscle excitation signals depending on the current foot-ground contact conditions, muscle states, and joint kinematics. We optimized the parameters of these control laws to simulate human-like walking on flat ground, as well as up inclines and with load. We first evaluated our simulation of normal walking by determining the percentage of joint angle and joint torque trajectories that were within 1 standard deviation of experimental data. In addition, we compared the timings of individual muscle force generation to EMG onset/offset timings. To determine if our simulations captured the salient adaptations to load and incline, we qualitatively compared predicted and experimental changes in kinematics, kinetics, muscle coordination, and energetics.

### Musculoskeletal Model

We adapted a two-dimensional (2D) musculoskeletal model described by Geyer and Herr [26]. The model had 9 degrees-of-freedom in the sagittal plane and 7 body segments (80 kg mass; 1.88 m height). A lumped trunk, head, and arms segment was connected to thigh segments for each limb by pin joints to represent the hip. The lower limbs consisted of thigh, shank, and foot segments, connected by knee and ankle joints, modeled as pin joints. The model was actuated by 8 Hill-type musculotendon units (MTUs) on each lower limb (ILPSO: iliopsoas; GMAX: gluteus maximus; HAMS: biarticular hamstrings; RF: rectus femoris; VAS: vasti; GAS: gastrocnemius; SOL: soleus; TA: tibialis anterior) (Fig. 1). The segment inertial parameters and joint limits were taken from Geyer and Herr [26], MTU model and parameters (i.e., moment arm curves, activation time constants, maximum isometric strength, optimal fiber length, and maximum shortening velocity) were based on the MTU model and values from Geyer et al. [36].

Unlike the model in Geyer and Herr [26], we include the RF muscle, raise the ankle position relative to the feet to better approximate human anatomy, and employ a different contact model. Contact with the ground plane was modeled using two spheres of radius 1 cm located at the heel and ball of the foot (Fig. 1). The normal contact force was generated using a compliant Hunt-Crossley model [37] when ground penetration (*h*) exceeded zero:

$${GRF}_{y}\;=\;kh^{1.5}\left(1+1.5c\dot{h}\right),$$

where *k* models the stiffness of the contact and *c* is a dissipation coefficient.

The friction force was given by

$${GRF}_{x}\;=\;\mu \left(\dot{x}\right){GRF}_{y},$$

where $\dot{x}$ is the tangential velocity between the contact point and the ground and *μ* is the coefficient of friction as a function of slip velocity $\left(\dot{x}\right)\;$ and the contact material. The material is fully defined by the static, dynamic, viscous coefficients of friction (*μ*_*s*,_ *μ*_*d*,_ *μ*_*v*_) and a transition speed (*v*_*t*_). We refer readers to Sherman et al. [38] for details.

The specific contact parameters we used were *k = 9*.*4281e5(Nm* ^-*1*.*5*^),*c = 2(m/s)* ^-*1*^, and *v*_*t*_ = *0*.*1(m/s)*,*μ*_*s*_ = *μ*_*d*_ = *0*.*8*_,_ *μ*_*v*_ = *0*.*5*. We tuned the contact parameters empirically for simulation speed while avoiding unrealistically “bouncy” surfaces. For example, we found that if the dissipation coefficient was too high, it caused the integrator to take small timesteps and thus slow simulations. The dynamics were enforced through forward simulation using Simbody/OpenSim [5, 38]. The same contact parameters were used for all simulations.

### MTU Feedback Controllers

Our control formulation was based on a combination of lower-level MTU feedback laws motivated by spinal reflexes and higher-level gait modes that depended on the phase of locomotion [26, 27]. In particular, excitation signals to the MTUs were determined by a combination of feedback control laws (Fig. 1), grouped into three categories: force feedback (*u* ^*F*^), stretch feedback (*u* ^*L*^), and PD control (*u* ^*θ*^). A simple first order activation dynamics model [26] was then used to generate activation inputs for MTU force generation. The specific laws for each MTU changed depending on the gait modes. We provide a high-level overview here and refer readers to Wang et al. [27] for details. Force feedback controllers excited each muscle in proportion to its normalized force (*F*_*MTU*_), delayed by D seconds:

$$u^{F}\;=\;G_{F}F_{MTU}\left(t-D\right).$$

Stretch feedback controllers excited each muscle in proportion to the muscle’s normalized fiber length (*l*_*CE*_) beyond a threshold H, delayed by D seconds:

$$u^{L}\;=\;G_{L}{\left[l_{CE}\left(t-D\right)-H\right]}_{+}.$$

The notation [*x*]_*+*_ for the PD and stretch feedback controller indicates that the signal is zero for,*x<0* and set to *x* otherwise. PD controllers were used to coordinate multiple MTUs to control angular features (*θ*). For example, the hip muscles and hamstrings work together to control the trunk orientation with respect to the ground during stance. PD controllers were also used for the hip and knee angles during the stance preparation gait mode (see below). PD controllers were defined as:

$$u^{\theta }\;=\;{\left[K_{p}(\theta \left(t-D\right)-\theta_{desired})+K_{d}\dot{\theta }\left(t-D\right)\right]}_{+}.$$

The muscle gain parameters *G*_*F*_,*G*_*L*_,*K*_*p*_,*K*_*d*_, the threshold parameter *H*, and the desired feature target *θ*_*desired*_, were free variables used by the optimizer to tune the controllers. Following Geyer and Herr [26], sensory feedback signal propagation delays were fixed at *D* = 5 ms for the ILPSO, GMAX, HAMS and RF; 10 ms for the VAS; and 20 ms for the GAS, SOL, and TA.

Each muscle had its own set of parameters for the MTU feedback laws and the laws for each muscle could change as the limb progressed through 3 gait-modes. Stance and swing modes were triggered by the foot making and breaking contact with the ground, respectively. We further divided the swing mode into “early swing” and “late swing”, which gave our model the flexibility to determine the landing configuration of the swing leg. Inclusion of the late swing mode was necessary for generating human-like inclined walking motions, since the early swing phase assumes the swing leg to be largely passive. The transition from early to late swing was triggered when the sagittal-plane distance between the limb ankle joint center and the model mass center was greater than a constant threshold d_SWING_, an additional parameter determined during the optimization. The control laws for each limb were symmetric (e.g., the right and left GMAX each had the same stance-phase PD controller).

### Objective Function

The objective of the optimization was given by:

$$R\;=\;w_{fail}J_{fail}+w_{vel}J_{vel}+w_{head}J_{head}+w_{effort}J_{effort}.$$

Specifically,

$$J_{fail}\;=\;\frac{1}{T_{fall}}(10-T_{fall}),$$

where *T*_*fall*_ is the first time in the simulation where the vertical COM drops below 0.7 m. Note that *T*_*fall*_ = *10* and *J*_*fail*_ = *0* if the model did not fall for the entire duration of the simulation. In practice, this term serves to terminate the simulation when the model has fallen down, which can significantly shorten the computing time during early iterations of the optimization. The next term is

$$J_{vel}\;=\;\frac{1}{\left|C\right|}\sum_{t\in C}Q({forward\_vel}_{t}-1.5,\;0.05),$$

where *Q(d*,*ɛ) = d* ^***2***^ if |*d*|>*ɛ*, *0* otherwise. *C* was the set of times when heel-strike occurred during the simulation, and *forward_vel*_*t*_ was the average forward velocity of the COM during the previous step computed at time *t*. This term captured the basic requirements of upright, forward progressing locomotion targeted at 1.5 m/s. Note that *ɛ = 0*.*05* m/s was an error threshold, such that we considered all solutions between 1.45 m/s and 1.55 m/s to have been sufficiently close to the target and were not penalized by *J_vel_*.

Additionally, the objective included

$$J_{head}\;=\;\frac{1}{\left|S\right|}\sum_{t\in S}Q({head\_vel}_{t},\;0.2),$$

where *S* was the set of timesteps sampled at 100 Hz (i.e., 0 s, 0.01 s, 0.02 s, etc.), and *head_vel*_*t*_ was the relative forward velocity between the head (top of the trunk segment) and the COM at time *t*. *J*_*head*_ was motivated by the observed desire of humans to stabilize the visual and vestibular systems during locomotion [39]. Lastly, the objective included the term

$$J_{effort}\;=\;\frac{1}{(M_{body}+M_{back})\left|S\right|}\sum_{t\in S}{Metabolics}_{t}+0.005{HPE}_{t},$$

where *M*_*body*_ and *M*_*back*_ were the mass of the body (80 kg) and backpack load, respectively. *Metabolics*_*t*_ was the total muscle metabolic power (J/s) at time *t*, *HPE*_*t*_ was the hyperextension penalty, defined as the total squared joint limit torque at time *t*. Readers are referred to Anderson and Pandy [17] and Wang et al. [27] for the precise definitions of *Metabolics*_*t*_ and *HPE*_*t*_.

We empirically set *w*_*fail*_ = *500000*, *w*_*vel*_ = *50*, *w*_*head*_ = *25*, *and w*_*effort*_ = *1*. The value of *w*_*fail*_ was large to reject any solutions where the model fell. The term, *w*_*vel*_ was set so that stable gaits achieving the target velocity could be quickly discovered. The head stability and effort terms were not as heavily weighted and were tuned to generate a human-like walking style on flat ground. Note that no further tuning was done for load and incline changes; the same weights were used for all simulations in this study.

### Predictive Simulation of Normal Walking

The movement predicted by our algorithm (i.e., the musculoskeletal simulation) was parameterized by a total of 74 design variables: 56 MTU controller parameters and the swing phase threshold distance d_SWING_ as defined above, along with 18 initial conditions for the joint angles and joint velocities. Optimization was used to solve for these design variables using a Covariance Matrix Adaptation (CMA) evolution strategy [40], which were initialized from previous work [26, 27]. Note that the initialization did not achieve stable walking due to differences between our foot and contact model and prior work. Simulations of 10 seconds were executed in parallel on a 48-node computing cluster (Amazon EC2). CMA is a gradient-free algorithm that adapts a Gaussian distribution (mean and covariance matrix) towards low energy regions. The covariance matrix is estimated by design variable candidate samples that can be evaluated in parallel. A master node containing the CMA optimization algorithm was responsible for generating and distributing sets of samples to each of the 48 slave nodes and evaluating the cluster of objective function values following an iteration of parallelized simulations. We took the mean stride cycle across the 10-second simulation as model prediction, ignoring the first two strides to allow the control excitation cycles to stabilize.

To evaluate our nominal predictive walking simulation, we obtained experimental unloaded, level walking data collected during a previous study [10]. These data came from a cohort of nine subjects (5 males, 4 females; age, 27.7 ± 8.0 years; mass, 73.1 ± 8.6 kg; height, 176 ± 7 cm; leg length 93 ± 5 cm) who walked at 1.5 m/s along a level platform while marker-based kinematics, ground reaction forces, and electromyography (EMG) data were simultaneously collected. Using a variant of the standard OpenSim model [4, 41] described in Dorn et al. [10], we performed inverse kinematics, inverse dynamics, and static optimization (with an objective to minimize the sum of all squared muscle activations) on the experimental data to obtain a time history of sagittal plane joint angles, joint torques, and muscle forces, respectively during a full cycle of gait. The computation was performed in OpenSim [5], independent from the predictive simulations described above to ensure an unbiased comparison of key walking variables from our predictive simulations. The reader is referred to Dorn et al. [10] for a complete description of the experimental protocols and analysis procedures.

We then compared the experimental joint kinematics, ground reaction forces, inverse dynamics-derived joint moments, static optimization-derived MTU forces, and muscle onset/offset timings (from EMG) to our simulation. We computed normalized kinematic, kinetic, and ground reaction force trajectories over a gait cycle and determined the percentage of the predicted curves that were within the equivalent experimental data. To assess muscle coordination, we compared the timing of muscle force generation in the simulation to corresponding muscle activation timing from EMG. The EMG onset/offset timings were determined by applying a Teager-Kaiser energy filter [42] to the raw EMG data sampled at 1500 Hz. We manually identified “off” regions for each muscle to estimate the corresponding noise distributions. A threshold of six standard deviations for onset detection was used as suggested by Li et al. [42].

### Predictive Simulations of Loaded and Inclined Walking

We generated simulations for level-ground walking with loads of 10%, 20%, 30% and 40% of body mass, denoted by *M*_*back*_ (i.e., 8–32 kg), and incline walking without load of 5°, 10°, 15°, and 20° (i.e., 8.7–36.4% grade). All achieved the same walking velocity of 1.45 m/s. The loads were modeled as an additional mass attached to the model’s torso 0.1 m posterior to the torso COM, with a moment of inertia of *0*.*15 M*_*back*_ (kg•m^2^). Separate optimization problems were solved for each of the loaded and incline scenarios, initializing each optimization to the solution of the previous optimization. For example, the optimized parameters from the level, unloaded walking simulation were used as the initial conditions to the optimization in which 10% of bodyweight (BW) was added; the optimized parameters from the 10% BW simulation were used as the initial conditions to the 20% BW optimization and so on. Optimizations for the inclined walking scenarios were performed in a similar fashion. We evaluated the predicted simulations by comparing against the experimental data captured by Silder et al. [34, 35] and Lay et al. [32, 33]. In particular, we examined whether the changes in metabolic cost, and peak joint angles, moments, muscle activity, and ground reaction forces in response to load or incline for our predictive simulation qualitatively agreed with the changes observed in these experimental studies.

## Results

All optimization scenarios found stable solutions with walking speeds of 1.45 ± 0.03 m/s. The objective function value for normal walking decreased rapidly in the first 1000 iterations, after which it decreased more slowly until iteration 2000, when we terminated the algorithm. The optimization took approximately 12 hours. Objective values for the loaded and incline walking cases stabilized more quickly as they were initialized from previous solutions. In these cases, we terminated the optimization when the objective values stopped improving for approximately 300 consecutive iterations. Visualization of these results is provided in the supplemental video (S1 File).

### Simulations of Normal Walking

**Fig 2:**
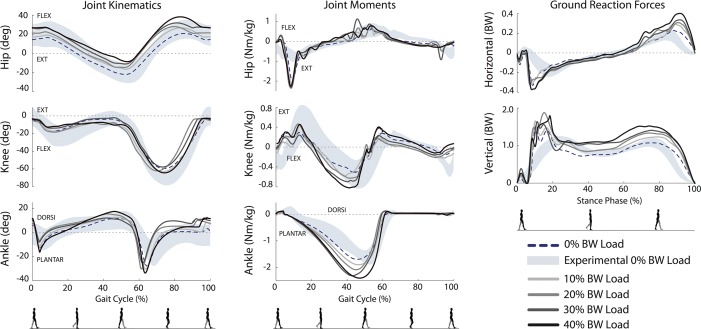
Sagittal plane joint kinematics, moments, and ground reaction force for each loaded walking condition. Experimental data for normal (unloaded) walking are represented by the shaded region (±1 SD). The dotted line represents equivalent simulation results for normal walking used to evaluate the model. Predictions of the loaded walking conditions are denoted by the solid lines.

Simulated level-ground, unloaded walking joint angles and moments were generally in agreement with human walking (Fig. 2). More specifically, 92% of the gait cycle of joint angles (hip 100%, knee 82%, ankle 95%) and 78% of the joint moments (hip 64%, knee 86%, ankle 83%) were within 1 standard deviation of experimental data. One notable exception was a higher ankle plantarflexion moment generated in the simulation. Ground reaction forces for normal walking displayed the typical biphasic profile except in the first 20% of stance, where impact spikes were present in the simulation, but not experimental data. A concomitant spike was observed in the hip extension moment immediately after heel-strike (Fig. 2, middle), likely to maintain trunk orientation in the presence of spikes in ground reaction forces.

**Fig 3:**
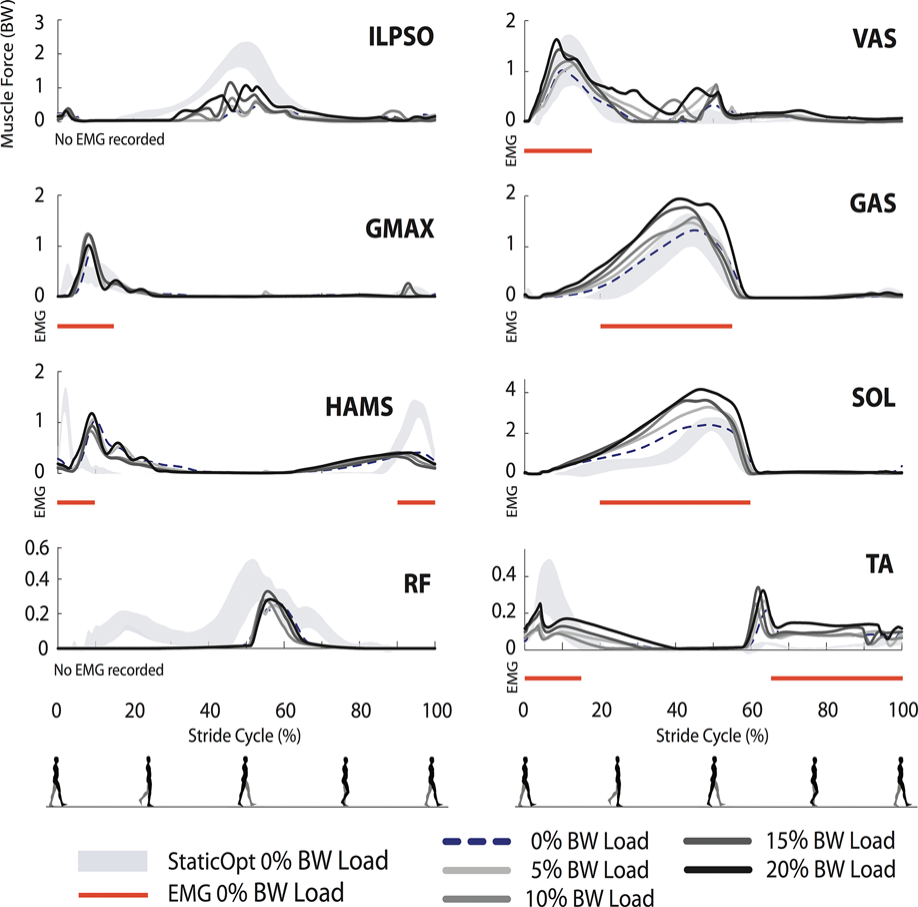
Simulated muscle forces for each loaded walking conditions. Static optimization-based muscle forces for normal (unloaded) walking are represented by the shaded region (±1 SD). The dotted black lines represent the muscle forces for normal walking predicted by our model. Predictions of muscle forces during loaded walking are denoted by the solid black and gray lines. Red lines correspond to periods of muscle activation from EMG data of normal walking.

**Fig 4:**
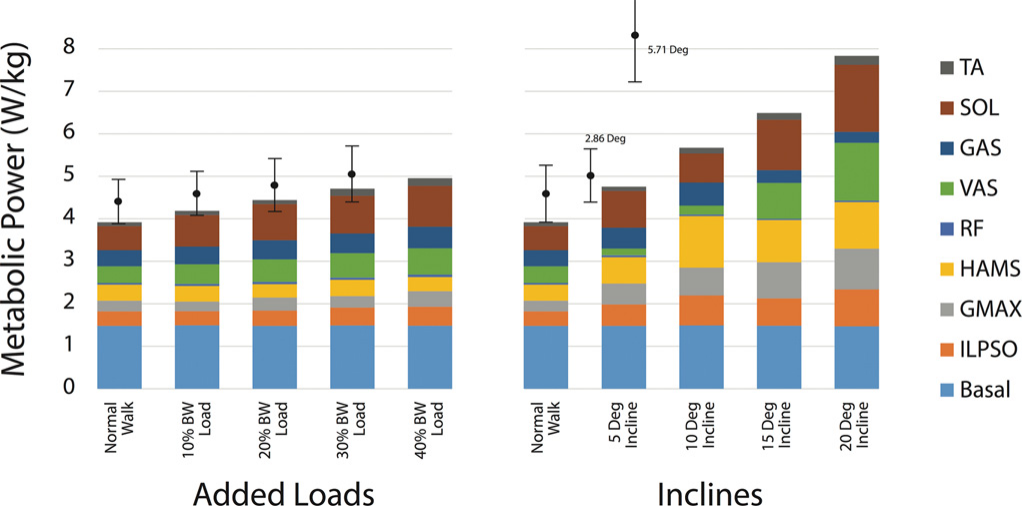
Metabolic power expenditure of the musculoskeletal model for each walking scenario. Net model expenditure is reported, normalized to unloaded body mass. Error bars on the left and right indicate ±1 SD of experimentally measured metabolic power from Silder et al. for loaded [35] and inclined walking, respectively [34]. The experimental mean and standard deviations are computed from the sum of the basal and gross metabolic costs (W/kg). The gross metabolic costs for loaded walking are weighted averages of the male and female data [35].

Simulated muscle force trajectories were similar in magnitude and timing to previous static optimization results, with the biggest differences present in the RF muscle in early stance, ILPSO in late stance, and HAMS in late swing (Fig. 3). Where available, the EMG onset/offset timings (Fig. 3, red bars) coincided with the generation of forces predicted by static optimization and therefore the trajectories from our predictive simulations as well, with the exception of HAMS. Note that when comparing force to EMG timings, we do expect there to be a small electromechanical delay. Our simulated metabolic energy expenditure (including basal rate) was 3.91 W/kg, which is approximately one standard deviation lower than the mean in both data sets reported by Silder et al. [34, 35] (Fig. 4).

### Simulations of Loaded Walking

Walking simulations across all loads predicted a constant stride length (1.45 m), which is consistent with experimental data. The metabolic cost increased with load, as in experiments (Fig. 4 left). The estimated values were smaller than in experiments, but still within one standard deviation of the experimental values reported by Silder et al. [35].

As load increased, the changes in kinematics were small and the model’s walking posture become slightly more crouched (Fig. 2 left), which is consistent with experiments [35]. Peak knee extension, knee flexion, and ankle plantarflexion moments during stance increased with load, which is also consistent with experiments. Increases were observed for the peak vertical ground reaction force in the 2^nd^ half of stance (Fig. 2 right). Contrary to the observation by Silder et al. [35], our percent increase in peak ground reaction force in the 2^nd^ half of stance roughly corresponded to the added load (with a rate of 9.8% for every load increase of 10% body weight).

Several muscles exerted greater peak forces in response to carrying increasing loads (Fig. 3), including VAS, GAS, and SOL which were muscles observed to have increasing peak muscle activities in EMG experiments [35]. We saw some increase in swing-phase HAMS forces for the heaviest load, although the trend was not as clear as the increase in peak hamstring EMG activities observed in experiments. We also saw increased GMAX and ILPSO forces with load. Experimental EMG for walking with load wasn't collected for these muscles, but the increased forces in these muscles are in agreement with the larger hip extension moment observed in loaded walking experiments.

### Simulations of Inclined Walking

**Fig 5:**
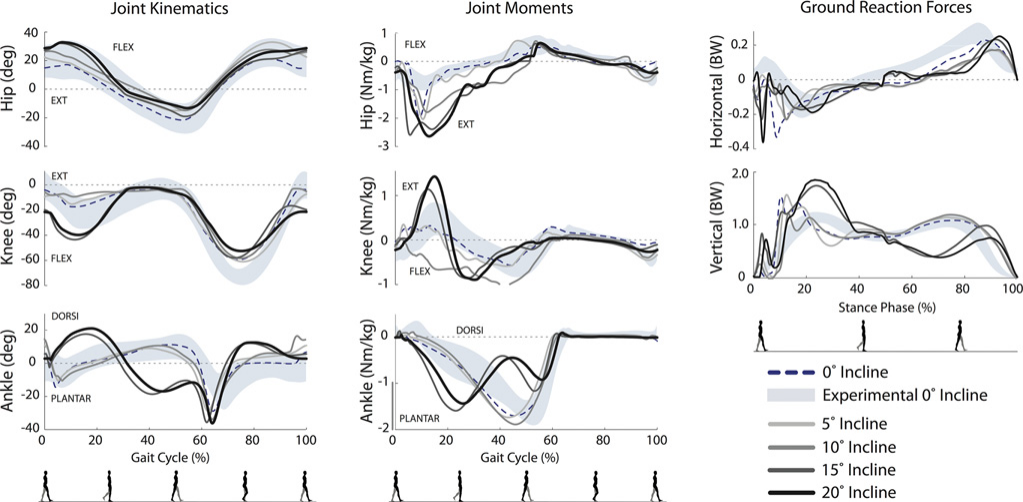
Sagittal plane joint kinematics, moments, and ground reaction force for each inclined walking condition. Experimental data for normal (0° incline) walking are represented by the shaded region (±1 SD). The dotted line represents equivalent simulation results for normal walking used to evaluate the model. Predictions of the inclined walking conditions are denoted by the solid lines.

Our model exhibited two distinct strategies as platform incline increased (Fig. 5). At an incline of 5° and 10°, the model adopted a strategy that was kinematically similar to normal walking, with hip, knee, and ankle curves largely within 1 standard deviation of normal gait. In contrast, inclines of 15° and 20° brought about a more crouched posture at ground contact as a consequence of planting the foot on a slope. This crouched gait was characterized by peak stance-phase hip flexion angles up to 33°, stance-phase knee flexion angles up to 42° and stance-phase ankle dorsiflexion angles up to 19°. A crouched posture was observed by Silder et al. [34] at lower inclines and Lay et al. [32] for larger inclines.

At higher inclines, our model predicted features not observed by Lay et al. [32] including plantarflexion in the second half of swing, and reduced stride length combined with increased stride frequency as inclines increased. Our predicted normalized metabolic cost increased at a substantially lower rate than experimental data. Specifically, Silder et al. [34] reported a mean increase (excluding basal rate) of 113% above level walking at 5.71° incline (Fig. 4 right). In contrast, our simulations predicted an increase of 34% at 5° and 71% at 10° incline above level walking. As in Silder et al. [34] and Lay et al. [32], the peak hip and knee extension and knee flexion moments in stance generally increased with incline, though in our simulations, we did not observe a clear increase in peak ankle plantarflexion moment and the 10° incline case showed knee flexion moments throughout stance.

**Fig 6:**
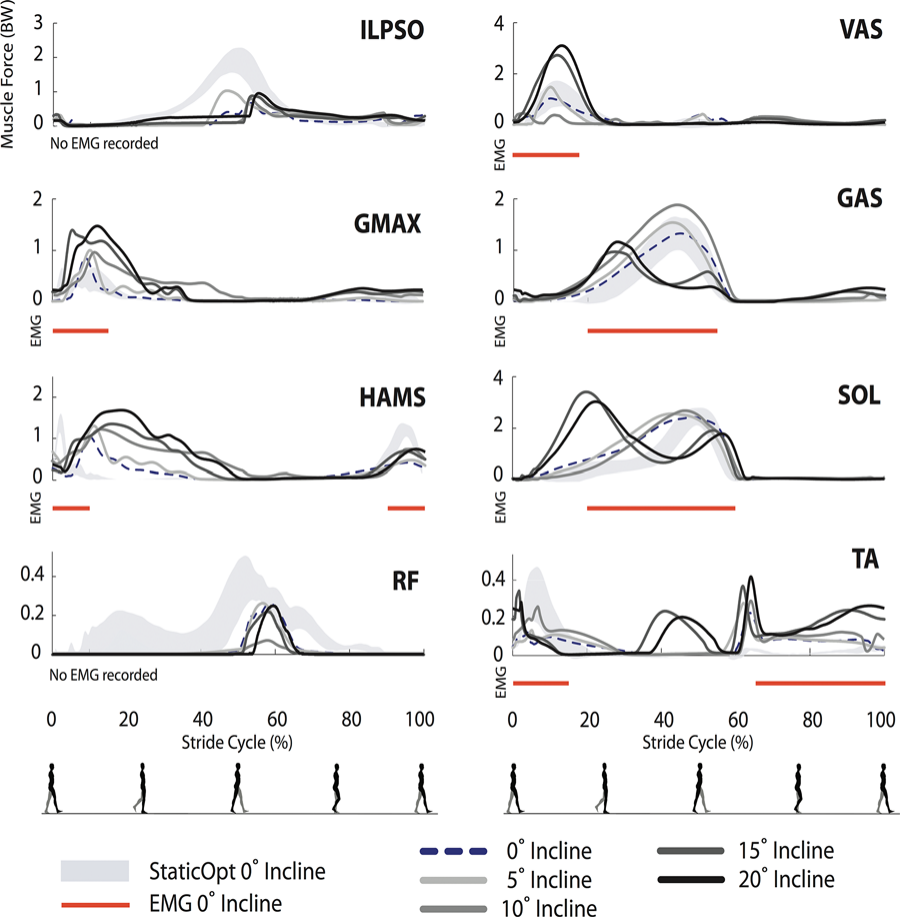
Simulated muscle forces for each inclined walking scenario. Static optimization-based muscle forces for normal (0° incline) walking are represented by the shaded region (±1 SD). The dotted black lines represent the muscle forces for normal walking predicted by our model. Predictions of muscle forces during inclined walking are denoted by the solid black and gray lines. Red lines correspond to periods of muscle activation from EMG data of normal walking.

For the low platform inclines (5° and 10°), the general shape of muscle forces did not change substantially from the level walking case. However, for the 10° incline, sustained force generation by HAMS along with corresponding knee flexion torques were seen throughout the stance phase. This was different from level walking and the 5° incline case. For the higher inclines, peak forces developed by HAMS, VAS, and GMAX increased (Fig. 6), which is consistent with changes in EMG activity from experiments [33, 34]. In our simulations, ILPSO, RF, and TA did not show any clear changes with incline. The forces developed in the ankle plantarflexors, SOL and GAS began to develop a double-peaked pattern for the higher inclines; SOL peaks also increased.

## Discussion

We generated predictive simulations of loaded and inclined human walking in a variety of scenarios to evaluate the capacity of our neuro-musculoskeletal model to synthesize motions adapted to different environments. Our results were generated using a combination of reflex control and effort minimization. We were able to reproduce the salient features of level, unloaded gait using a relatively simple musculoskeletal model. In particular, our kinematic, joint moment, and ground contact force results were mostly within 1 standard deviation of experimental data, and the onset/offset timing of muscle forces closely matched experimentally-determined EMG recordings. Our model was also able to adapt to walking with a load of up to 40% bodyweight and inclines of up to 20°. While previous muscle-driven predictive simulations of walking have been evaluated against experimental unloaded walking data on flat ground [7, 17, 19, 25–27, 31], our predictive model is the first to compare synthesized kinematics, kinetics, muscle coordination, and metabolic adaptations to experiments with increasing load and incline.

### Normal Walking

Our result for walking on flat ground matched both kinematics and kinetics measured experimentally, to a degree comparable with state-of-the-art in predictive gait simulation [19, 25–27, 31]. We also reproduced the general lower limb muscle force coordination pattern and vertical ground reaction force profile from human walking.

A notable difference between our simulations and experimental data was the sharper spike in ground reaction force upon heel-strike, which is likely to produce large knee loads and cause discomfort in human subjects. Incorporating additional terms such as joint loading in the objective function could encourage a strategy with a smoother initial heel-strike. Note the sharp spike has been observed in prior predictive simulation of gaits [7, 19, 23, 25, 26] and could also be an artifact of the simplified contact models typically employed in predictive simulation. Improving the ground contact model to better match experimental force profiles is an important area of future work.

### Loaded Walking

For loaded walking, as in experimental studies of human subjects, the general strategy for locomotion, including kinematics and muscle coordination did not change with increasing load [35]. In particular, stride length and stride frequency were invariant, and in general, joint angles stayed close to unloaded gait kinematics. Contrary to the data presented by Silder et al. [35], we observed that an increase in added load as a percent of bodyweight leads to an almost identical percent increase in peak ground reaction force during late stance. Adding a joint loading term to the cost function, in addition to effort minimization, might reduce the ground reaction forces in our simulations of loaded walking.

As load increases, greater body support and extension moments are required. The knee extension and ankle plantarflexion moments increased with load, along with the muscle forces from VAS, GAS, and SOL (Fig. 3). These changes in force generation agree with the increased muscle activities observed experimentally [35] and prior studies showing that the vasti and plantarflexors are the primary muscles that extend the joints and support the body during walking [43]. We observed an increase in metabolic cost with load, as in experiments, with the support muscles VAS, GAS, and SOL, accounting for the majority of this increase.

### Inclined Walking

Our relatively simple musculoskeletal model and neural control framework was also able to synthesize biologically feasible walking up to a 20° incline (see S1 File). Walking on a steep incline required a crouched strategy with increased hip and knee flexion, along with greater stance-phase dorsiflexion, as observed experimentally [32, 34].

There was also some divergence between model and experiment. The predicted motion for the 10° incline exhibited insufficient knee flexion during stance despite increased knee flexion moments and hamstring force outputs. Moreover, the model reduced stride length and increased stride frequency, in contrast with experiments by Lay et al. [32]. One hypothesis to explain this discrepancy is that humans may have a preferred stride length and frequency on inclines, even if it is not metabolically optimal. We also observed that for small changes in incline (5° and 10°) our simulations used a kinematic, kinetic, and muscle force pattern closer to level walking than in experiments. It should also be noted that our simulations required a target velocity of 1.45 m/s, which is faster than speeds in experimental studies (0.8 to 1.2 m/s). Further experimental study, at faster speeds, fixed stride lengths and frequencies would help resolve these questions. As discussed in the previous section, our prediction of metabolic cost with increasing incline was also significantly lower than experimental data. Exploring alternative models of muscle metabolic energy expenditure [25] or even alternative objectives for locomotion [19] are interesting future directions.

### Limitations and Opportunities for Future Work

Our normal walking predictions show differences from human ground truth data, which may be due to limitations of the present study. First, while the reflex-based controller we employ is biologically motivated, it is necessarily only a crude approximation of the actual control mechanism used in human locomotion. One potential direction for future work is to rigorously analyze the biological basis of the parameters used in our control algorithm. For example, we would like to examine the control gains recovered in the optimization process as well as the feedback signal transmission delays assumed by our model. The latter, for example, is somewhat short compared to experiments [44]. It would also be fruitful to explore non-reflex based contributions to muscle activation, such as from central pattern generators [45].

Second, we employed a simplified 2D sagittal plane musculoskeletal model for our analysis. Although human walking is predominantly a sagittal plane motion, additional degrees of freedom in the coronal and transverse planes allow the pelvis to rotate around the stance leg contributing to a longer stride [46] and permit the ankle to rotate providing additional ground clearance for the foot during the swing phase [47]. Models with coronal and transverse plane muscles would permit three-dimensional predictive simulations and provide the capability to simulate pathological gaits that are characterized by significant out-of-plane motion [48, 49]. While 3D muscle-driven predictive models exist [17, 31], future studies could investigate if explicit reflex control laws can be developed for non-sagittal muscles and if they can improve the quality of the predictions.

In addition, the non-convex nature of our optimization problem prevents us from asserting that our predicted gait simulations are globally optimal. The results we presented correspond to locally low-energy solutions, which could vary due to different initializations and the stochastic nature of the CMA algorithm. Exploring the sensitivity of solutions to these factors is an interesting direction for future work.

### Conclusions

The predictive simulation approach presented in this paper was able to synthesize motions with both visual and biomechanical fidelity for a range of loads and inclines. Improving the reliability of predictive simulation and validating predictions in more scenarios remain crucial topics for research. Once the accuracy of predictive simulations are appropriately tested and validated, the simulations provide a powerful framework for studying adaptations in kinematics, kinetics, and muscle coordination in a broad range of applications, from assistive device design to surgical planning.

## Supporting Information

S1 File Supplemental Video. (MP4)
